# Nanocomposite Treatment Reduces Disease and Lethality in a Murine Model of Acute Graft-versus-Host Disease and Preserves Anti-Tumor Effects

**DOI:** 10.1371/journal.pone.0123004

**Published:** 2015-04-13

**Authors:** Priscila T. T. Bernardes, Bárbara M. Rezende, Carolina B. Resende, Talles P. De Paula, Alesandra C. Reis, William A. Gonçalves, Elias G. Vieira, Maurício V. B. Pinheiro, Danielle G. Souza, Marina G. M. Castor, Mauro M. Teixeira, Vanessa Pinho

**Affiliations:** 1 Laboratório de Resolução da Resposta Inflamatória, Departamento de Morfologia, Instituto de Ciências Biológicas, Universidade Federal de Minas Gerais, Belo Horizonte, Minas Gerais, Brazil; 2 Laboratório de Interação Microorganismo e Hospedeiro, Departamento de Microbiologia, Instituto de Ciências Biológicas, Universidade Federal de Minas Gerais, Belo Horizonte, Minas Gerais, Brazil; 3 Laboratório de Ressonância Paramagnética, Departamento de Física Instituto de Ciências Exatas,Universidade Federal de Minas Gerais, Belo Horizonte, Minas Gerais, Brazil; 4 Laboratório de Imunofarmacologia, Departamento de Bioquímica e Imunologia, Instituto de Ciências Biológicas, Universidade Federal de Minas Gerais, Belo Horizonte, Minas Gerais, Brazil; Charité, Campus Benjamin Franklin, GERMANY

## Abstract

Graft versus host disease (GVHD) is an immunological disorder triggered by bone marrow transplantation that affects several organs, including the gastrointestinal tract and liver. Fullerenes and their soluble forms, fullerols, are nanocomposites with a closed symmetrical structure with anti-inflammatory and anti-oxidant properties. The present study evaluated the effects of treatment with the fullerol (C60(OH)18-20) in the development and pathogenesis of GVHD in a murine model. Mice with experimental GVHD that were treated with the fullerol showed reduced clinical signs of disease and mortality compared with untreated mice. Treatment with the fullerol decreased the hepatic damage associated with reduced hepatic levels of reactive oxygen species, pro-inflammatory cytokines and chemokines (IFN-γ TNF-α, CCL2, CCL3 and CCL5) and reduced leukocyte accumulation. The amelioration of GVHD after treatment with the fullerol was also associated with reduced intestinal lesions and consequent bacterial translocation to the blood, liver and peritoneal cavity. Moreover, the fullerol treatment alleviated the GVHD while preserving effects of the graft against a leukemia cell line (GFP+P815). In summary, the fullerol was effective in reducing the GVHD inflammatory response in mice and may suggest novel ways to treat this disease.

## Introduction

Allogeneic bone marrow transplantation is the only curative therapy for hematological malignancies. However, GVHD is a severe and often fatal complication following allogeneic HSCT and is the major cause of morbidity in up to 50% of transplant patients [1, 2]. GVHD is characterized by immunosuppression, activation of APCs, production of inflammatory mediators [3–5] and intense recruitment and activation of immune cells accounting for damage to target organs including the liver, intestine, skin and lung [6–8]. Currently, various therapies to treat GVHD are available, such as the use of high doses of steroids, monoclonal antibodies targeting the host APCs and immunosuppressive agents [8, 9]. However, while reducing GVHD in patients, these treatments increase the possibility for immunosuppression and the incidence of infection and contribute to morbidity and mortality [10].

Fullerenes are a broad family of symmetrical nanomolecules formed by hexagons interconnected by pentagons, the latter being responsible for the curvature of the molecule and consequently for its three-dimensional shape and were discovered in 1985 by Kroto [11] and collaborators and have attracted attention because of their biological and pharmacological properties. Fullerol is the most abundant and representative soluble form of fullerenes derived from C60 (C_60_(OH)_18–20_) and is formed by the addition of polar groups (OH) [12]. Fullerol has been utilized as an effective intervention for the control of the inflammatory responses in some experimental models by reducing the levels of inflammatory cytokines and oxidative stress [13–17]. Additionally, it also has potential applications as a pharmaceutical for the treatment of several other illnesses [18].

In this study, we hypothesized that treatment with fullerol in GVHD might control the inflammatory response leading to the prevention of acute graft versus host disease. To test this hypothesis, we evaluated the effects of treatment with the fullerol in mice with experimental GVHD.

## Materials and Methods

### Ethics Statement

The animal care and handling procedures were in accordance with the guidelines of the Institutional Animal Care and Use Committee, and the study received prior approval from the local animal ethics committee (Animal Ethics Review Board—Comitê de Ética em Experimentação Animal-CETEA/Universidade Federal de Minas Gerais-UFMG (protocol number: 209/2011). Animals judged to be moribund were euthanatized with an overdose of anesthesia (mixture of ketamine (37.5 mg/ml, final concentration) and xylazine (2.5 mg/ml, final concentration) and counted as GVHD lethality. At the end of these experimental procedures, the remaining mice were also euthanized with an overdose of anesthetics. In all experiments the efforts were made to minimize suffering at all times.

### Mice

Eight- to 12-week-old C57BL/6 and B6D2F1 (C57BL/6 X DBA/2) mice were obtained from the Centro de Bioterismo (UFMG) and housed under standard conditions in a temperature-controlled room (23±1°C) on an automatic 12-h light/dark cycle. The mice had free access to commercial chow and water. The number of mice in each specific group is provided in the figure legend. One representative experiment with at least 5 mice per group of similar independent experiments is shown in each figure.

### Fullerol (C_60_(OH)_18–20_)

The fullerenes, being hydrophobic, require functionalization with polar groups for most biologic applications. One of the simplest ways of making fullerenes hydrophilic is by attaching multiple hydroxyl groups to the fullerene carbon cage, resulting in a polihydroxy-fullerene salt known as a fullerol or fullerenol. In this work, we synthesized this salt by means of a phase transfer reaction using tetrabutylammonium hydroxide (TBAH) as a catalyst. The original synthesis using polyethylene glycol as the phase transfer catalyst is described elsewhere [19]. For this work, we modified the synthesis procedure as follows. Initially, 22 g of NaOH was dissolved in 40 mL of deionized water. In parallel, 36 mg of the C_60_ fullerenes (99.5% Sigma Aldrich) was added to 40 mL of toluene. This aqueous solution was stirred constantly and heated to 50°C before 0.1 mL of TBAH was added. The fullerene organic solution was concomitantly added dropwise to the aqueous solution. After the reagent solutions were mixed, the final solution was stirred for 3 hours. At the end of this procedure, two phases were clearly observed: the first, which was colorless, was the remaining toluene after the phase transfer, whereas the second, a brownish-red sludge, was the alkaline aqueous phase with the fullerenes carried by the TBAH. The colorless organic phase was then removed by evaporation, and then, the remaining solution with the brown sludge was reduced to 30 mL with the help of a rota-evaporator and filtered under vacuum with a Millipore system. The filtered alkaline aqueous solution, which had a brownish red color and was cleared of the sludge, was further reduced to 20 mL and had a pH of 14. The last part of the process involved the reduction of the pH with multiple washings with methanol. First, 20 mL of the brown alkaline aqueous solution was diluted in 500 mL of methanol for 10 min of stirring, and then, it was vacuum filtered with a further dilution of the material retained in the filter in 20 mL of water. This process was repeated several times until the pH of the aqueous solution was reduced to 7. At the end, the remaining solution was dried in an oven for 8 hours and quality controlled by infrared spectroscopy. The resulting brown powder was a fullerol salt similar to [C_60_(OH)_x_O_y_]^n-^[Na_n_]^n+^ (x = 12 to 15, y = 7 to 9 and n = 2 to 3) [20]. In this synthesis, approximately 1 g of the fullerol was produced. The composition was, however, nearer to the commercial C_60_(OH)_18–22_(OK)_4_ (F2), with Na^+^ replacing the K^+^ as the counter ions [21]. For simplicity, we refer to the fullerol used in this work as C_60_(OH)_18–20_.

### Induction of GVHD

#### Low dose regimen

GVHD was induced by i.v. injection of 3×10^7^ splenocytes from syngeneic (B6D2F1) or semiallogeneic (C57BL/6) donors into recipient B6D2F1 mice that had been irradiated with a single 4 Gy dose of low body irradiation (source CO^60^) 2 days prior to transplantation, as described previously [3–5,7].

#### High dose regimen

Recipient B6D2F1 mice were irradiated with 8 Gy of total body irradiation (source CO^60^) in 2 doses at a 2 hour interval to minimize gastrointestinal toxicity, followed by an i.v. infusion of 3×10^7^ splenocytes and 1×10^7^ bone morrow cells from syngeneic (B6D2F1) or parent (C57BL/6) donors. In this model, due to the toxicity of the high body irradiation, the recipient mice received an oral suspension of ciprofloxacin (70 mg/L) in their drinking water from 1 day before to 15 days after the transplantations [7].

#### Cell preparation

Bone marrow suspensions were prepared by flushing the mouse femurs with RPMI 1640 medium that was supplemented with 10% FCS. The splenocytes were obtained by gently crushing the spleens in complete medium to release the cells, which were then filtered to remove the debris and washed twice in PBS before the injections.

### Therapies

The fullerol was given at a dose of 10 mg/kg (C60 (OH) 18–20), i.p. in 100 μL of PBS, 30 minutes before the transplant and then every 48 hours until the end of experiments. In the same way, the GVHD group was treated with 100μL PBS (i.p) every 48 hours. The control group (B6D2F1 to B6D2F1) did not receive a treatment.

### Mortality rate and assessment of GVHD clinical score

The mice were monitored daily for survival after the transplants and were evaluated clinically with a standard scoring system that generated a composite GVHD score from the individual scores for weight loss, posture (hunching), activity, fur texture, skin integrity, diarrhea, and fecal occult blood. A clinical index was subsequently generated by summing the 7 criteria scores (maximum index = 14), as described previously [3–5].

### Histopathology

A set of experiments was conducted for the quantification of histopathological parameters in the liver and small intestine. Tissues sections were processed for histological analysis as described previously [3] and were evaluated by a pathologist who was blinded to treatment groups. A numerical value was attributed to the changes observed in these organs, and each animal received a score generated by summing the changes observed (maximum index, 6 for the liver and 9 for the intestines) [3]. Histopathological scores were assigned to the samples obtained from the mice at 10 and 20 days after the transplant, which corresponds to the early clinical signs and the mortality phase in the mice, respectively.

### Bacterial translocation

At day 20 after the transplantation, 100 μl of blood, 100 μl of peritoneal lavage fiuid and 100 mg of liver homogenate were plated onto Muller Hilton plates. These plates were incubated for 24 h at 37°C and the numbers of bacterial colonies were counted and expressed as the CFUs.

### Quantification of macrophage infiltration

The relative numbers of infiltrating macrophages in the intestine and liver were quantified by measuring the NAG activity (N-acetyl-β-D-glucosaminidase activity) on day 20 after the transplantation. A 100 mg portion of the small intestine was resuspended in 0.9% saline (4°C) with 0.15% v/v Triton X-100 (Merck, Rahway, NJ, USA), homogenized, and centrifuged at 4°C for 10 min at 1500 rpm. The supernatants were collected and assayed immediately for NAG activity at a 1:10 dilution as described previously [22].

### Quantification of neutrophilic infiltration

The relative number of neutrophils infiltrating into the liver was quantified by measuring the MPO activity (myeloperoxidase activity) at 10 and 20 days after the transplant. A portion of the 100 mg of the liver was removed and frozen at -70°C for later analysis. Upon thawing, the tissue (0.1 gm of tissue per 1.9 ml of buffer) was homogenized and processed for the determination of the MPO activity as described previously by Souza [23]. The assay used 25 mL of 3,3 ′-5,5′- tetramethylbenzidine (TMB, Sigma, St Louis, MO, USA) in PBS (pH 5.4) as the color reagent.

### Quantification of cytokines and chemokines

The concentrations of cytokines and chemokines were quantified from the intestinal or liver homogenates from the animals at days 3, 10, and 20 after transplantation. The tissues were mixed with PBS that contained antiproteases (0.1 mM PMSF, 0.1 nM benzethonium chloride, 10 mM EDTA, and 20 KI aprotinin A) and 0.05% Tween 20. Next, the samples were centrifuged for 10 min at 10,000 rpm and 4°C. Dilutions of the supernatants in PBS (1:4) were immediately analyzed by ELISA. The cytokine and chemokine concentrations were measured according to the manufacturer’s procedures (R&D Systems, Minneapolis, MN, USA) and the colorimetric reactions were analyzed with a spectrophotometer at a wavelength of 492 nm.

### Reactive oxygen species (ROS) analysis

ROS production was assessed by fluorescence 20 days after transplantation in the liver and was measured using the ROS-specific fluorescent probe H2 DCFDA 2'-7’- dichlorofluorescein diacetate (DCF-DA) as described previously by Amaral [24]. Briefly, suspension of cells from the liver was incubated with the DCF-DA probes (20 mM) for 30 minutes in an incubator at 37°C in the dark because the markers are photosensitive. The reading of the fluorescence was performed in a spectrophotometer (Synergy 2, Biotek) at an excitation wavelength of 485 nm and with emission at 530 nm. The data are expressed as the mean fiuorescence.

### GVL induction

A P815 mouse mastocytoma cell line (H-2^d^; American Type Culture Collection, Manassas, VA, USA) that had been transduced with a lentiviral vector (elongation factor 1-GFP) was kindly provided by Anna C. Leal and Martin Bonamino (Instituto Nacional do Câncer, Rio de Janeiro, Brazil). This cell line was maintained in RPMI/10% FCS at 37°C and 5% CO_2_ and was used for the GVL (graft-versus-leukemia) experiments *in vivo* [4,5]. The above-described protocols for irradiation and GVHD induction were used. The B6D2F1 recipients were injected i.v. with 10^4^ GFP+ P815 cells on day 0 of the transplantation experiment. After the induction of GVHD and the transplantation of tumor cells, the mice were monitored every 2 days for survival. The fullerol group was treated with fullerol (C60 (OH) 18–20), i.p. in 100 μL of PBS every 48 hours beginning one day before the transplantation and continuing until the end of the experiment. At the end of the experimental procedure, the remaining mice were euthanized with an overdose of anesthesia.

### Confocal microscopy

At Day 10 after the transplant, mice were killed and lymphoid organs (lymph node and spleen) prepared for confocal microscopy analysis. Images were obtained using C2 Eclipse Ti confocal microscope (Nikon). Total cell counts were performed in a modified Neubauer chamber using Tripan’s stain. Tumor cells (GFP+P815 cell) were identified in cytocentrifuged slides, which were also stained with DAPI to nuclear stain. Fluorescence intensity was measured off-line using Volocity software 6.3 (Perkin-Elmer) and the fluorescence profile was assessed using Image J (NIH).

### Statistical analysis

The data in the text are expressed as the mean ± SEM. Comparisons between the groups were performed by ANOVA, followed by a Student Newman-Keuls *post hoc* analysis. A log-rank test was used to compare the relevant survival curves. Statistical significance was set at p<0.05, and the graphs were created and the analyses were performed with GraphPad Prism 4 software (GraphPad Software Inc., San Diego, CA, USA).

## Results

### Treatment with fullerol prevents mortality and morbidity in mice with experimental GVHD

**Fig 1:**
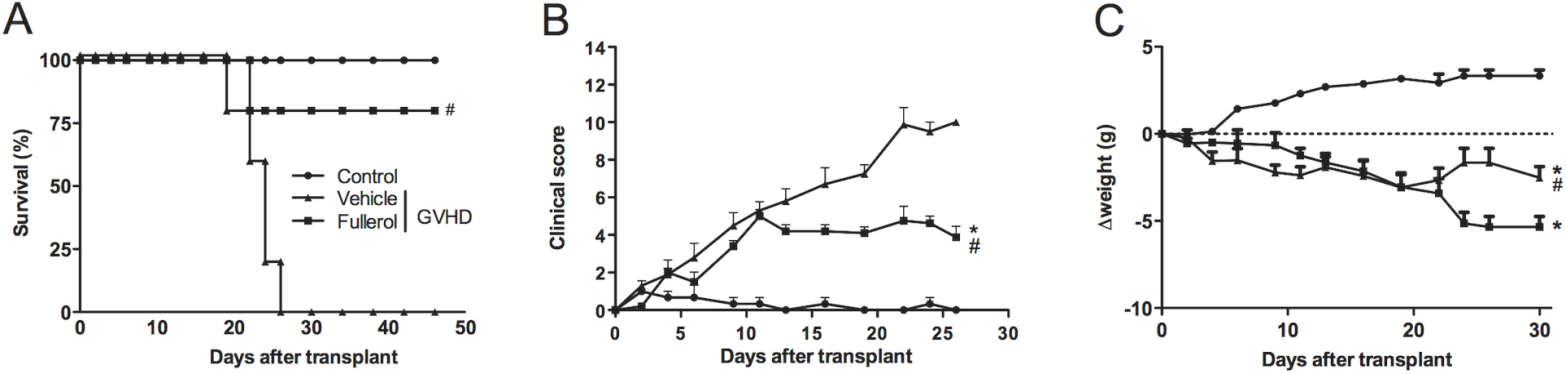
Fullerol treatment reduces mortality and clinical signs in experimental GVHD. GVHD was induced by the transfer of 3 x 10^7^ splenocytes from C57BL/6J donors to B6D2F1 mice that were irradiated with low dose two days before transplantation. The mice that received splenocytes from the syngeneic (B6D2F1) mice did not develop the disease and were considered the control group. Fullerol (10 mg/Kg in 100 ml of PBS, i.p.) was given to the WT mice 30 minutes before the transplantation and every 48 hours thereafter during the 28 days of the experiment (period during which all of the animals from the GVHD group died). After the induction of GVHD, the mice were monitored every 2 d for survival (A), GVHD clinical scores (B) and body weight (C). The results are shown as the mean ± SEM, and the number of animals was as follows: Control group (●, n = 5); GVHD group (▲, n = 6); fullerol group (■, n = 6). * and # P < 0.05 compared with the control and GVHD groups, respectively.

Initial experiments were performed to evaluate the effects of fullerol treatment on the survival and clinical signs in murine GVHD, as described in the Methods section. In the model using low body irradiation, which induces a GVHD less severe providing a more accurate evaluation of inflammatory parameters and the effects of treatments [7], the transplant of splenocytes from C57BL/6 donors to recipient B6D2F1 mice (GVHD group) resulted in 100% lethality by day 26 after the disease induction. In contrast, the mice with experimental GVHD treated with fullerol (fullerol group) exhibited 80% survival (Fig 1A). The fullerol treatment was terminated after all the animals from the GVHD group died. These mice remained alive until day 47, the last day of the observation (Fig 1A). The control group did not develop GVHD, and thus, all of these mice were alive at the end of the experiment. Consistent with the higher survival observed in the GVHD fullerol-treated mice these animals had lower clinical scores compared with the untreated GVHD group: absence of occult blood in feces, diarrhea, hunching (posture) and apathy and preservation of skin integrity and fur texture (Fig 1B). At day 22 after transplantation, fullerol treatment significantly ameliorated weight losses. (Fig 1C).

To confirm the effects of the fullerol treatment on GVHD and to establish the relevance to human disease, we tested the fullerol treatment in mice with experimental GVHD using a high dose of irradiation that results in a more severe disease and that involves bone marrow transplantation. The irradiation dose is proportional to the degrees of tissue damage and cytokine production, and these factors can influence the development of GVHD [7]. We observed that the transplant of splenocytes and bone marrow from the C57BL/6 donor to the recipient B6D2F1 mice (GVHD group) resulted in 100% lethality and higher clinical scores and weight loss 21 days after the disease induction (Panel A-C in S1 Fig). The fullerol treatment prolonged survival, reduced the clinical scores and resulted in lesser weight loss (Panel A-C in S1 Fig). At 21 days after the transplant, the treatment was interrupted and the survival was 67% until the end of experiment. The remaining mice were killed on day 40, the last day of the observation (Panel A in S1 Fig). To determine whether fullerol treatment could be associated with undesired effects in the mice, the animals without experimental GVHD were treated with fullerol for 20 days. Our results show that the nanocomposite had no effect on body weight and the survival of the mice (data not shown).

### Treatment with the fullerol ameliorates GVHD-related hepatic damage

**Fig 2:**
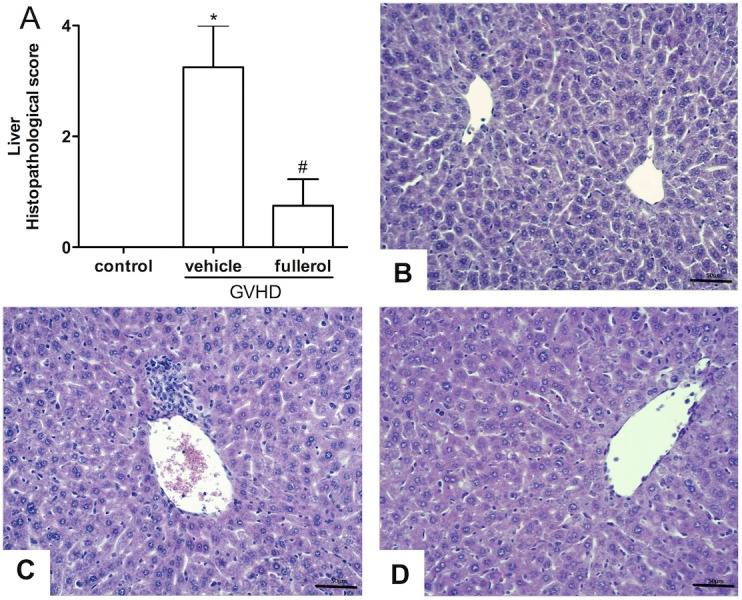
Fullerol treatment reduces hepatic injury in mice with experimental GVHD. GVHD was induced by the transfer of splenocytes from semi allogeneic C57BL/6J donors to B6D2F1 mice. Fullerol (10 mg/Kg in 100 ml of PBS, i.p.) was given to the B6D2F1 mice 30 min before transplantation and every 48 hours thereafter during the entire duration of the experiments. The mice that received splenocytes from the syngeneic (B6D2F1) mice did not develop the disease and were considered the control group. After the induction of GVHD, the mice were killed and the liver sampled for histopathological analysis and scoring at 20 days post-transplantation (A). Histological aspects of the H&E-stained liver sections in the control, GVHD, and fullerol treated mice, respectively (B-D). The scale bar = 50 μm for all the panels. The results are presented as the mean ± SEM (n = 6); * and #, P < 0.05 when compared with the control and GVHD groups, respectively.

**Fig 3:**
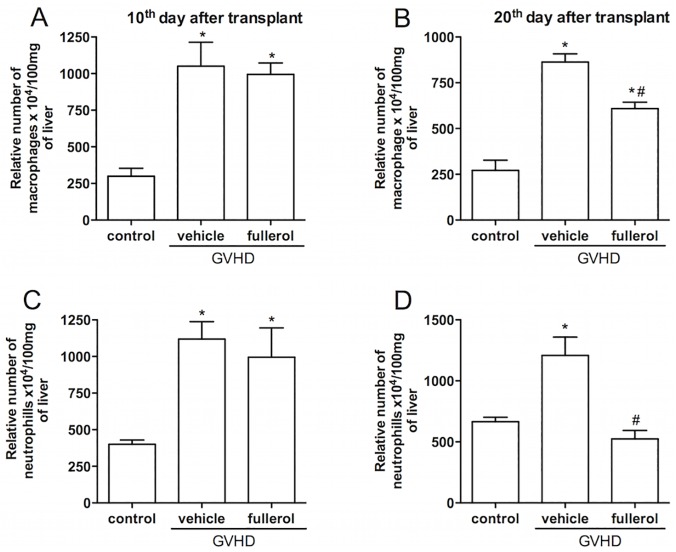
Fullerol treatment decreased the accumulation of leukocytes in the liver at 20 days after transplant. GVHD was induced by the transfer of splenocytes from semi allogeneic C57BL/6J donors to B6D2F1 mice. Fullerol (10 mg/Kg in 100 ml of PBS, i.p.) was given to the B6D2F1 mice 30 min before transplantation and every 48 hours thereafter during the entire duration of the experiments. The mice that received splenocytes from the syngeneic (B6D2F1) mice did not develop the disease and were considered the control group. Liver samples were collected 10 and 20 days after transplantation, and the accumulation of macrophages and neutrophils was analyzed in this tissue through the activity of N-acetyl-β-D-glucosaminidase (NAG) and myeloperoxidase (MPO), respectively. The relative numbers of macrophages 10 days (A) and 20 days (B) after transplantation are shown. The relative numbers of neutrophils 10 days (C) and 20 days (D) after transplantation are shown. The results are presented as the mean ± SEM (n = 6), * and #P < 0.05 compared with the control and GVHD groups, respectively.

Because the partial body irradiation GVHD model allowed a more detailed evaluation of the clinical signs of GVHD due to the reduced severity of the disease, the next experiments were performed in this model. Liver sections were processed at days 10 and 20 after the transplant, which corresponded to the onset of clinical signs and the mortality phase, respectively. At day 10, the GVHD group had a modest inflammatory cell infiltrate and some degenerative signs in the parenchyma and the periportal areas, and the fullerol treatment did not modify these parameters (data not shown). However, at day 20 after the transplantation, the GVHD group showed significant liver injury throughout the liver parenchyma and increased inflammatory infiltration mainly in the periportal areas (Fig 2B). Moreover, vasodilatation, hepatocyte necrosis and diffuse vacuolization were observed in this group (Fig 2B). In contrast, the fullerol group had lower hepatic histopathological scores (Fig 2A). This was characterized by a significant reduction in the observable degenerative process and infiammatory cell accumulation compared with the GVHD group (Fig 2B–2D). There were no histopathological changes in the control group at any time point. In addition, we quantified the accumulation of macrophages and neutrophils by the activity of N-acetyl-**β**-D-glucosaminidase (NAG) and myeloperoxidase (MPO), respectively. The GVHD group had increased macrophage and neutrophil accumulation compared with the control group at days 10 and 20 after the transplant (Fig 3A–3D). The fullerol treatment significantly reduced the accumulation of these cells at 20 days (Fig. 3B–3D).

### Treatment with fullerol decreased the levels of inflammatory mediators in the livers of mice with experimental GVHD

**Fig 4:**
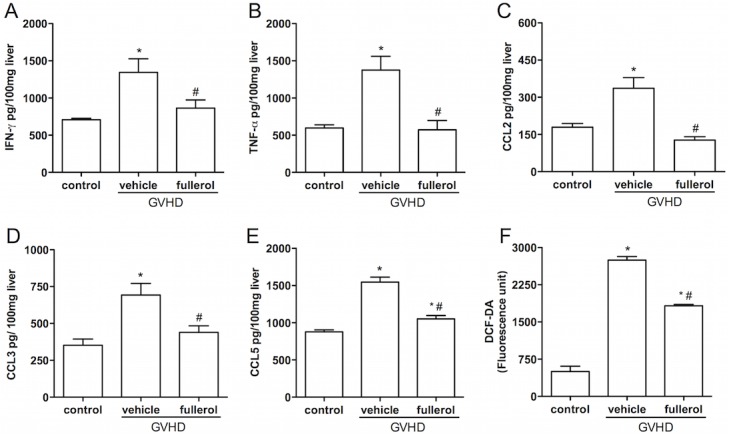
Fullerol reduces the concentration of cytokines, chemokines and reactive oxygen species in the liver. GVHD was induced by the transfer of splenocytes from semi-allogeneic WT or C57BL/6J donors to B6D2F1 mice. The mice that received splenocytes from the syngeneic (B6D2F1) mice did not develop the disease and were considered the control group. Fullerol (10 mg/Kg in 100 ml of PBS, i.p.) was given to the WT mice 30 min before transplantation and every 48 hours thereafter during the entire duration of the experiments. At 20 days after the induction of GVHD, the mice were killed, the concentrations of IFN-γ (A), TNF-α (B), CCL2 (C), CCL3 (D) and CCL5 (E) in the liver were evaluated by ELISA, and the levels of reactive oxygen species were evaluated by fluorescence units in the liver (F). The results are shown as the mean ± SEM (n = 6). * and #P <0,05 compared with the control and GVHD groups, respectively.

In agreement with results described above, at day 10 after the transplant, there was no difference between the levels of TNF-**α**, IFN-**γ**, CCL2, CCL3 and CCL5 in the mice with experimental GVHD regardless of whether they received treatment with fullerol (data not shown). At day 20, the treatment with fullerol reduced the levels of TNF-**α**, IFN-**γ**, CCL2, CCL3 and CCL5 to background levels (Fig 4A–4E).

The levels of ROS in the livers of the mice with experimental GVHD were increased on day 20. However, treatment with fullerol significantly reduced the levels of ROS (Fig 4F).

### Treatment with fullerol prevents bacterial translocation and intestinal injury in mice with experimental GVHD

**Fig 5:**
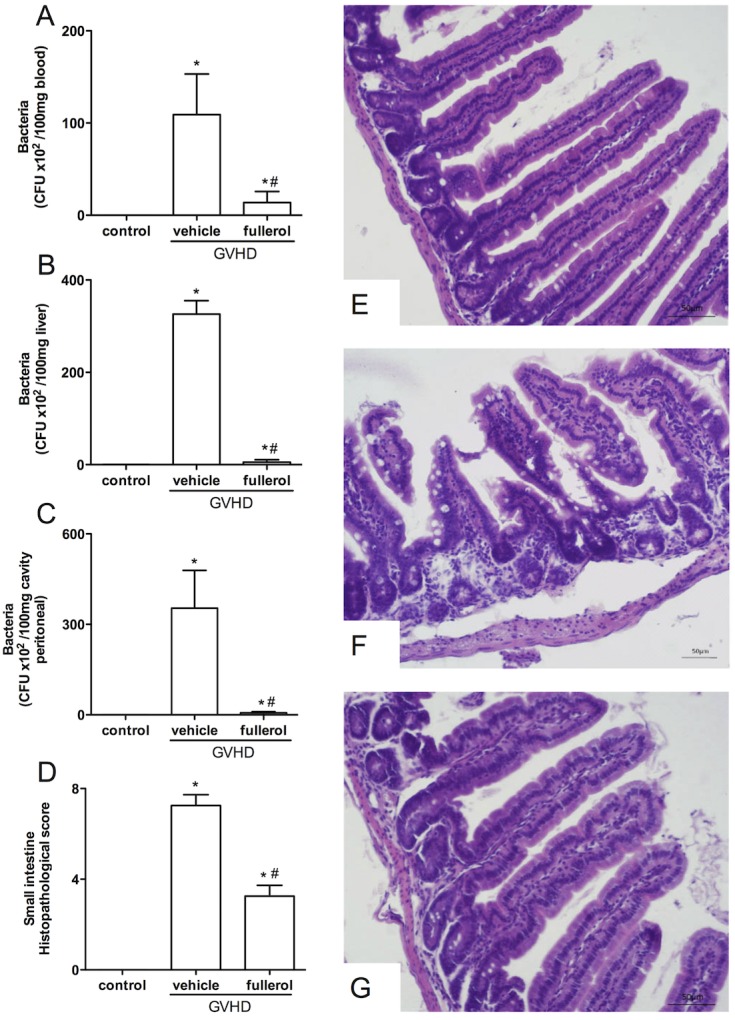
Fullerol treatment was associated with the inhibition of bacterial translocation and intestinal injury. GVHD was induced by the transfer of splenocytes from semi allogeneic C57BL/6J donors to B6D2F1 mice. Fullerol (10 mg/Kg in 100 ml of PBS, i.p.) was given to the B6D2F1 mice 30 min before transplantation and every 48 hours thereafter during the entire duration of the experiments. The mice that received splenocytes from the syngeneic (B6D2F1) mice did not develop the disease and were considered the control group. At Day 20 after transplant, the mice were sacrificed and bacterial translocation to the peritoneal cavity (A), blood (B), and liver (C) were quantified. The small intestine samples were also removed for histopathological analysis (D). The histological aspects of the H&E-stained small intestine sections in control, GVHD, and fullerol treated mice, respectively (E-G) The scale bar = 50 μm for all the panels. Control group (n = 5); GVHD group (n = 6); and fullerol group (n = 6). * and # *P* < 0.05 compared with the control and GVHD groups, respectively.

Bacterial translocation aggravates GVHD and can result in sepsis and patient death [25, 26]. At day 20, the numbers of bacterial colony forming units (CFUs) in the peritoneal cavity, liver and blood of the mice with experimental GVHD and treated with the fullerol were significantly reduced compared with the untreated mice (Fig 5A–5C). The reduced bacterial translocation in the mice with experimental GVHD and treated with the fullerol correlated with the preservation of the intestinal parenchyma. Indeed, there was a reduced infiammatory infiltrate, a milder edema in the lamina propria and the preservation of the muscular and serous layers, as well as a significant decreased in the degenerative process compared with the GVHD group (Fig 5D–5G).

### Treatment with fullerol decreased the GVHD but did not interfere with the GVL response in mice

Allogeneic hematopoietic stem cell transplantation is the main therapy used to eliminate hematological diseases by graft-versus-leukemia (GVL) activity by donor T-cells [1]. To verify whether fullerol interfered in the GVL response, graft versus host disease was induced by a transfer of allogeneic splenocytes from the C57BL/6J to recipient B6D2F1 mice. The control group received a transfer of syngeneic splenocytes from the B6D2F1 mice. The mice with experimental GVHD were treated with vehicle alone or with the fullerol. To verify the GVL response, the GVHD mice were injected with 10^4^ GFP+P815 cells immediately after the splenocyte transplantation (day 0). One group of the GVHD mice that received the GFP+P815 cells had been treated with fullerol. Finally, to verify the viability of the P815 cells, one group of the mice received only the P815 injection in the absence of experimental GVHD. The mice were monitored every 2 d for survival.

**Fig 6:**
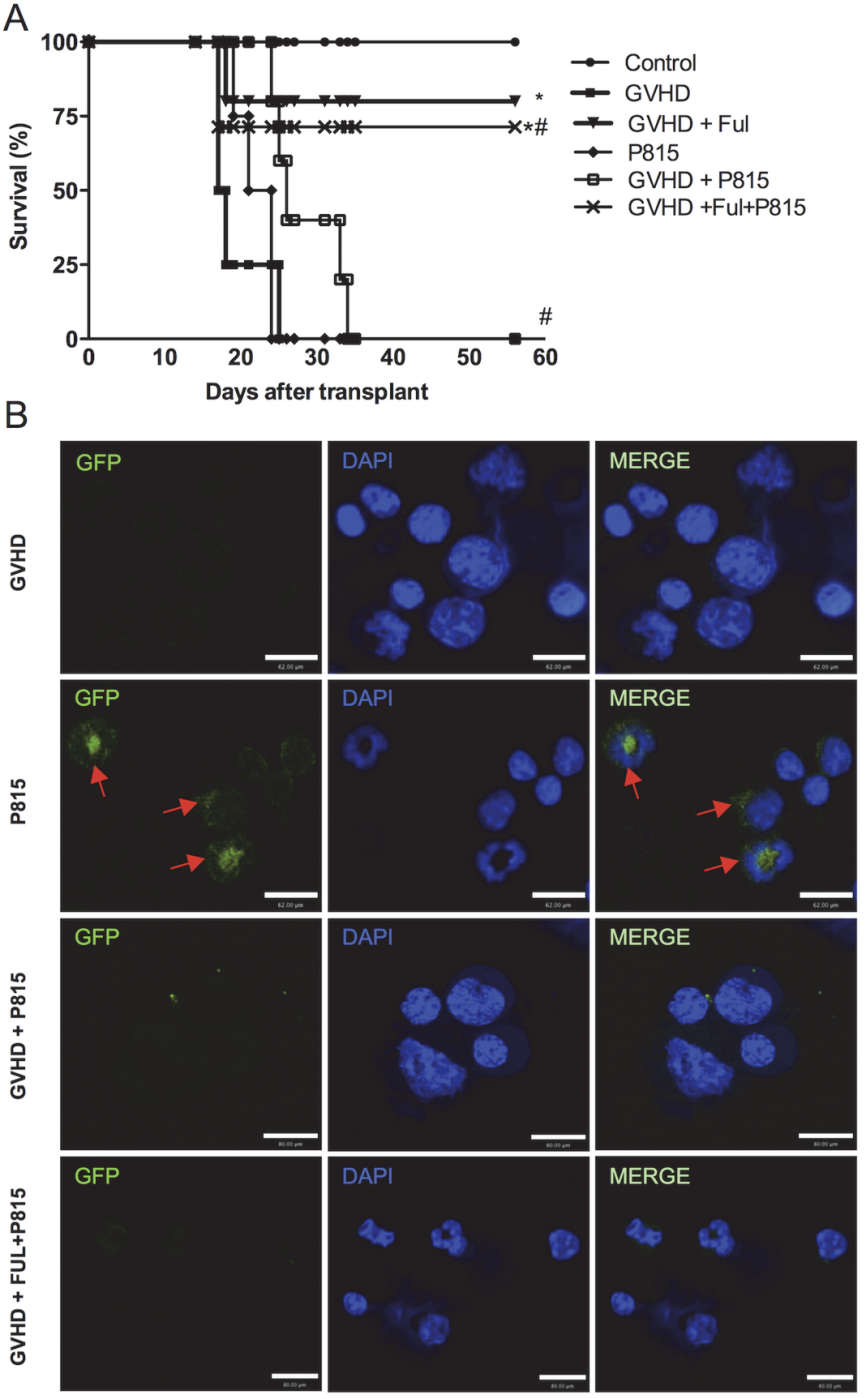
Fullerol treatment does not interfere with GVL in mice with experimental GVHD. GVHD was induced by the transfer of splenocytes from allogeneic (C57BL/6J) donors to B6D2F1 mice. GFP+P815 cells were injected i.v. into the B6D2F1 recipients on day 0 of transplantation. The mice that received splenocytes alone from the syngeneic (B6D2F1) mice did not develop the disease and were considered the control group. The P815 group received the GFP+P815 cells alone and the other groups received splenocytes from the allogeneic (C57BL/6J) donors and the GFP+P815 cells. Fullerol (10 mg/Kg in 100 ml of PBS, i.p.) was given to the WT mice 30 min before transplantation and every 48 hours thereafter during the entire duration of the experiments. After the induction of GVHD, the mice were monitored every 2 d for survival (A). The results are shown as the mean ± SEM (n = 6). In other experiment, GFP+P815 cells were evaluated in lymphoid organs by confocal microscopy (B). Arrows show GFP+P815. 60x. The scale bar: GVHD = 62 μm; P815 = 62 μm; GVHD + P815 = 80 μm and GVHD + P815 + ful = 80 μm. Red arrows show GFP+P815 tumor cells. * and # P <0,05 compared with the control and GVHD groups, respectively.

There were no deaths in the control group (mice that did not receive tumor cells and did not develop GVHD). The GVHD group that had not received the fullerol treatment (GVHD group) died before 25 days after transplant. The mice that received only the tumor cells (P815 group) all died by day 25 after transplantation, which indicated the viability of the tumor cells. The GVHD mice that received P815 (GVHD + P815 group) all died before 35 days after transplant. Thus, the GVHD mice that received the P815 cells died from GVHD rather than from the tumors. The fullerol treatment reduced the severity of the GVHD without interfering with the beneficial responses of the allogeneic cells against the tumors because the mice that received both the splenocytes and the tumor cells (GVHD + ful + P815) had a survival rate (71,4%) similar to the fullerol-treated mice that received only the splenocytes (80%) (GVHD+ful) (Fig 6A). In addition, there was a marked increase in the frequency of P815 in lymphoid organs (inguinal and mesenteric lymph node and spleen) of mice that were not subjected to GVHD (P815 group). In contrast, in mice subjected to GVHD there was a reduced frequency of GFP+P815 tumor cells in lymphoid organs, suggesting that there was a GVL effect. Treatment with fullerol also decreased the frequency of GFP+P815 tumor cells, suggesting that fullerol did not affect GVL (Fig 6B). These results support our hypothesis that mice not subjected to GVHD (transplant syngenic) died because of development of tumor and those subjected to allogenic transplant died because of GVHD.

## Discussion

Herein, we provide evidence for the application of fullerol in the treatment of murine GVHD. The major findings of the current study can be summarized as follows: 1) Fullerol treatment ameliorated the clinical signs of GVHD and improved survival; less hepatic damage was associated with decreased hepatic levels of TNF-α and IFN-γ and chemokines, including CCL2, CCL3 and CCL5, reactive oxygen species, and leukocyte accumulation was reduced in the mice with experimental GVHD that were treated with fullerol. 2) Amelioration of the GVHD after treatment with fullerol was also associated with reduced intestinal lesions and consequently reduced bacterial translocation. 3) Finally, the fullerol treatment alleviated the GVHD without impairing the beneficial effects of the graft against a leukemia cell line (GFP+ P815).

In our model, treatment with fullerol prevented death in the mice with experimental GVHD. The survival was associated with less injury in the liver. Hepatic dysfunction is a common complication of graft-versus-host disease [27, 28]. Of note, 50% of patients with GVHD have liver injury associated with hepatomegaly, abnormal levels of liver enzymes in the blood and hyperbilirubinemia [29, 30]. Here, we demonstrated that treatment with fullerol decreased liver injury. We further demonstrated that treatment with fullerol was correlated with reduced leukocyte accumulation and decreased levels of inflammatory cytokines (IFN-**γ** and TNF-**α)** and chemokines (CCL2, CCL3 and CCL5) in this organ. Corroborating our results, Yudoh and collaborators [17] demonstrated that the water-soluble fullerene (C60) reduced the levels of inflammatory cytokines such as TNF-**α** in a rat model of arthritis. Some cytokines and chemokines, such as IFN-**γ**, TNF-**α**, CCL2, CCL3 and CCL5, are well known to have relevant roles in GVHD [2–5, 31, 32]. It is known that there is a correlation between high levels of IFN-**γ** and TNF-**α** and the severity of GVHD [29, 32–34]. Chemokines such as CCL2, CCL3, and CCL5 are also important in the activation and recruitment of leukocytes to inflammatory sites and are associated with target organ damage in GVHD [31–33]. Terwey and colleagues [35] reported that the receptor for the cytokine CCL2 (CCR2) has an important role in the activation and migration of CD8+ T cells into the intestines and liver of animals with experimental GVHD. Therapies based on the blockade of CCL3 were effective to control murine GVHD by minimizing the clinical signs of disease and increasing the survival rate of the animals [3]. In 2007, Choi and colleagues [2] showed that the absence of CCR1 and its interaction with its ligand (CCL5) was related to greater survival of the mice with GVHD, reduced clinical signs of the disease and the reduced production of proinflammatory cytokines. Chemokines are important for the recruitment of leukocytes to inflammatory sites and their activation. These cells play an important role in GVHD, leading to the destruction of host tissues and the increased production of inflammatory mediators and this process is associated with the GVHD mortality [1, 31, 33]. Additionally, the fullerol treatment reduced the accumulation of macrophages and neutrophils in the liver. Thus, we believe that the effect of the fullerol in on reducing the levels of cytokine, chemokine and leukocyte accumulation in the liver become relevant to controlling the outcome of GVHD in mice. Hepatitic GVHD usually follows cutaneous and/or intestinal GVHD and may rapidly progress to ductopenia and deep jaundice [36].

Because fullerenes (C60) can act as an antioxidants [37–39], we investigated their effects on the ROS production in the target organs of GVHD. The antioxidant potential that is attributable to the fullerenes and their derivatives is as a result of the symmetrical and stable structure of these compounds, which makes them excellent electron acceptors; thus, fullerenes and their derivatives are very efficient at capturing free radicals and working as a "free radical sponge" [37]. Here, the capacity of fullerol to decrease ROS implies that its antioxidant effects may be associated with the control of the inflammatory response reflected in an improvement in the clinical signs and the increased survival of the mice with GVHD. Fullerol does not prevent release but removes ROS from the system. ROS are known to be inflammatory mediators inducer and there is an association between increase of reactive oxygen species and production of cytokines and proinflammatory chemokines [40]. Thus, reduction of proinflammatory mediators after treatment with fullerol appears to be secondary to decrease of ROS levels in sites of GVHD injury.

It is known that GVHD is also characterized by intestinal injury [41–44]. Intestinal injury is associated with bacterial translocation to the liver and subsequently into the bloodstream [25, 43]. Here, we showed a reduction in the intestinal damage associated with decreased bacterial translocation into the peritoneal cavity, blood and liver in the mice with experimental GVHD that were treated with fullerol. The capacity of fullerol to control the bacterial translocation is an important factor because sepsis is a major cause of death in patients with GVHD [25, 43].

Graft-versus-host disease is the main complication of hematopoietic stem cell transplantation; however, it may have significant antitumor benefits, often termed a graft-versus-leukemia (GVL) effect. Several therapies that decrease GVHD are frequently able to reduce GVL activity, which is the ability of donor-derived infused lymphocytes in a hematopoietic stem cell-transplanted patient to react against leukemic cells [44, 45]. The observation that GVL is maintained and that initial disease (see body weight and clinical score) is similar in treated and untreated mice suggests that the effects of fullerol is mostly on the effector phase of the immune response. Given this, reducing GVHD while preserving the GVL effects may be an interesting therapeutic strategy after HSCT. Of note, the fullerol treatment inhibited the GVHD but did not affect the ability to induce the GVL response.

It is worth noting that the protective effects of the fullerol are not associated with the toxic effects because the animals without experimental GVHD that were treated with fullerol did not show changes in weight and survival. The toxicity of the water-soluble fullerene has been the focus of some authors [38, 46, 47]. Gharbi and collaborators [38] studied the effects of C60-pretreatments on acute carbon tetrachloride intoxication in rats and showed that the aqueous C60 suspensions did not have acute or subacute toxicity in rodents. In addition, the fullerol does not exhibit appreciable toxicity, unless it is combined with ultraviolet light [39, 48].

In summary, the results presented in our study demonstrate that the fullerol treatment was effective in reducing the inflammatory response associated with GVHD without interfering with the graft versus leukemia response. Thus, the use of fullerol may be a promising strategy for the treatment of GVHD and different diseases associated with a persistent inflammatory response.

## Supporting Information

S1 Fig Fullerol reduces mortality and clinical signs in a model of severe GVHD. GVHD was induced by the transfer of 3x10^7^ splenocytes and 1x10^7^ bone marrow cells from semi-allogeneic WT or C57BL/6J donors to the B6D2F1 mice, which had been irradiated with a high dose for bone marrow depletion. The mice that received cells from the syngeneic (B6D2F1) mice did not develop the disease and were considered the control group. Fullerol (10 mg/Kg in 100 ml of PBS, i.p.) was given to the WT mice 30 min before transplantation and every 48 hours thereafter during the twenty days of the experiment (period during which all the GVHD group animals died). After the induction of GVHD, the mice were monitored every 2 d for survival (A), GVHD clinical scores (B) and body weight (C). The results are shown as the mean ± SEM (n = 6). * and # P < 0.05 compared with the control and GVHD groups, respectively.
(TIFF)
