# Supplementary figures and images for: Nanocomposite Treatment Reduces Disease and Lethality in a Murine Model of Acute Graft-versus-Host Disease and Preserves Anti-Tumor Effects
Source: PLoS One. 2015 Apr 13;10(4):e0123004. doi: 10.1371/journal.pone.0123004 (PMC4395348; doi:10.1371/journal.pone.0123004)

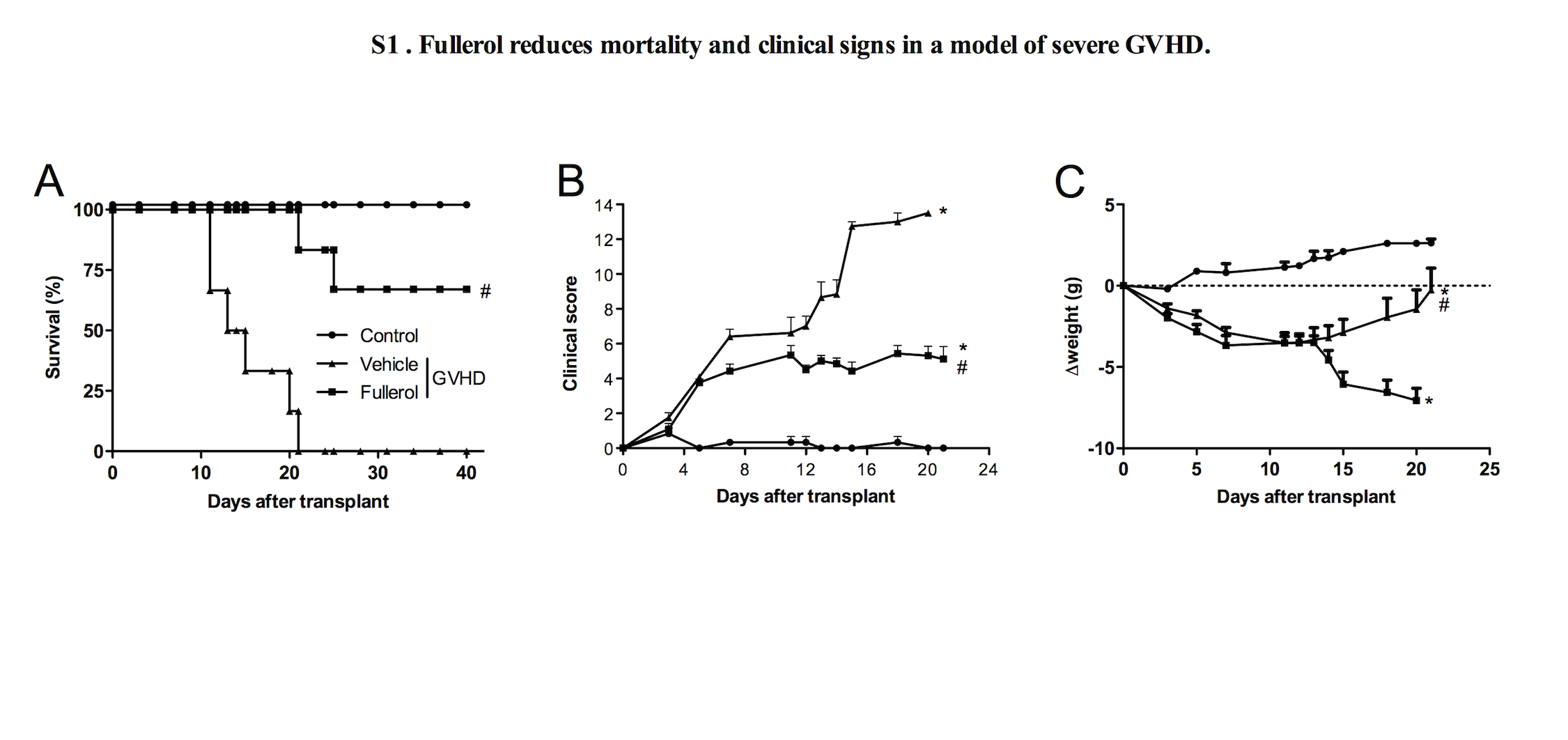

Supplement: S1 Fig — GVHD was induced by the transfer of 3x10^7^ splenocytes and 1x10^7^ bone marrow cells from semi-allogeneic WT or C57BL/6J donors to the B6D2F1 mice, which had been irradiated with a high dose for bone marrow depletion. The mice that received cells from the syngeneic (B6D2F1) mice did not develop the disease and were considered the control group. Fullerol (10 mg/Kg in 100 ml of PBS, i.p.) was given to the WT mice 30 min before transplantation and every 48 hours thereafter during the twenty days of the experiment (period during which all the GVHD group animals died). After the induction of GVHD, the mice were monitored every 2 d for survival (A), GVHD clinical scores (B) and body weight (C). The results are shown as the mean ± SEM (n = 6). * and # P < 0.05 compared with the control and GVHD groups, respectively. (TIFF) [file pone.0123004.s001.tiff]
